# Proteomic signatures of schizophrenia-sourced iPSC-derived neural cells and brain organoids are similar to patients' postmortem brains

**DOI:** 10.1186/s13578-022-00928-x

**Published:** 2022-12-01

**Authors:** Juliana Minardi Nascimento, Verônica M. Saia-Cereda, Giuliana S. Zuccoli, Guilherme Reis-de-Oliveira, Victor Corasolla Carregari, Bradley J. Smith, Stevens K. Rehen, Daniel Martins-de-Souza

**Affiliations:** 1 Laboratory of Neuroproteomics, Department of Biochemistry and Tissue Biology, Institute of Biology, University of Campinas (UNICAMP), Rua Monteiro Lobato, Campinas, SP 255, 13083-862 Brazil; 2 D’Or Institute for Research and Education (IDOR), Rua Diniz Cordeiro, 30, Rio de Janeiro, RJ 22281-100 Brazil; 3 Department of Biosciences, Institute Science and Society, Federal University of São Paulo (UNIFESP), Santos, SP Brazil; 4 Institute of Biology, Federal University of Rio de Janeiro (UFRJ), Rio de Janeiro, RJ Brazil; 5 Instituto Nacional de Biomarcadores Em Neuropsiquiatria (INBION), Conselho Nacional de Desenvolvimento Científico E Tecnológico (CNPq), São Paulo, Brazil; 6 Experimental Medicine Research Cluster (EMRC), University of Campinas, Campinas, SP 13083-970 Brazil

**Keywords:** Neuroproteomics, Schizophrenia, Brain organoids, Neural cells, Human pluripotent stem cells

## Abstract

**Background:**

Schizophrenia is a complex and severe neuropsychiatric disorder, with a wide range of debilitating symptoms. Several aspects of its multifactorial complexity are still unknown, and some are accepted to be an early developmental deficiency with a more specifically neurodevelopmental origin. Understanding the timepoints of disturbances during neural cell differentiation processes could lead to an insight into the development of the disorder. In this context, human brain organoids and neural cells differentiated from patient-derived induced pluripotent stem cells are of great interest as a model to study the developmental origins of the disease.

**Results:**

Here we evaluated the differential expression of proteins of schizophrenia patient-derived neural progenitors (NPCs), early neurons, and brain organoids in comparison to healthy individuals. Using bottom-up shotgun proteomics with a label-free approach for quantitative analysis, we found multiple dysregulated proteins since NPCs, modified, and disrupted the 21DIV neuronal differentiation, and cerebral organoids. Our experimental methods have shown impairments in pathways never before found in patient-derived induced pluripotent stem cells studies, such as spliceosomes and amino acid metabolism; but also, those such as axonal guidance and synaptogenesis, in line with *postmortem* tissue studies of schizophrenia patients.

**Conclusion:**

In conclusion, here we provide comprehensive, large-scale, protein-level data of different neural cell models that may uncover early events in brain development, underlying several of the mechanisms within the origins of schizophrenia.

**Supplementary Information:**

The online version contains supplementary material available at 10.1186/s13578-022-00928-x.

## Introduction

Schizophrenia is one of the most prevalent neuropsychiatric disorders, though poorly understood regarding its molecular mechanisms. In general, initial symptoms are present at the beginning of adulthood; however, molecular roots of the disease have been linked to neurodevelopmental dysfunctions [1]. Several symptoms overlap with other disorders, such as major depression and bipolar disorders, making an early diagnosis and treatment strategies more difficult [2]. Both genetics and the environmental course on neurodevelopment have been associated with the disease onset. Thus, continued discoveries and characterization of new factors that contribute to disease onset and progression are key to understanding the complexity of the disorder [3].

Advances over the past few years are a result of employing a broader spectrum of tools to research *postmortem* brains and animal models, from brain imaging to cell-based studies, including induced pluripotent stem cells (iPSCs) [4, 5]. Furthermore, human iPSCs (hiPSCs) overcome the impracticality and poor accessibility of human brain cell types [6] and are providing the possibility to challenge and question neural cell fates, prior to or during the onset of a disease in question. Considerable progress has been made regarding the neural differentiation of human pluripotent stem cells into mature neurons and cerebral organoids [7]. Human neural progenitor cells (hNPC) form a useful cell system for high-throughput screening due to their homogeneity, along with low complexity and limited differentiation potential. In comparison, brain or cerebral organoids are complex, three-dimensional (3D) culture systems composed of multiple cell types that self-organize into various brain regions similar to those in vivo, including the cerebral cortex, ventral forebrain, midbrain-hindbrain boundary, and hippocampus [8–10]. The combination of different cell types in a complex 3D configuration can better simulate brain biology and function, allowing cerebral organoids to reproduce the function and architecture of the brain, especially regarding development and neuronal plasticity.

Through the combination of hiPSCs with neural organoid differentiation and label-free shotgun proteomics, we can expand our knowledge of expression dynamics, providing a deeper understanding of biological processes along the course of development, and simulating in vivo conditions [5, 11]. Therefore, here we evaluate schizophrenia using neural progenitor cells (NPCs), immature neurons, and brain organoids derived from schizophrenia patients and compared them to control subject-derived neural cells using shotgun label-free proteomics. Cells derived directly from schizophrenia patients offered an integrated view of protein expression during neurodevelopment, depicting compromised biochemical pathways. In addition, it established an in vitro schizophrenia platform with the potential to modify and manipulate compromised pathways using a plethora of chemical compounds. This preliminary global proteome analysis during cell development may contribute to the search for new therapies with a focus on the personalized treatment of the disease.

## Materials and methods

### Pluripotent stem cell culture

Human induced pluripotent stem cells (hiPSCs) were reprogrammed from three subjects diagnosed with the schizophrenia spectrum, available at Coriell [Cat# GM23760, RRID:CVCL_T804 (male, 26y, paranoid schizophrenia); Cat# GM23761B, RRID:CVCL_T807 (female, 27y, schizoaffective disorder); and Cat# GM23762B, RRID:CVCL_T808 (male, 23y, early onset schizophrenia)] [12]. Three control hiPSC cell lines were used, a Coriell Cat# GM23279A, RRID:CVCL_F178 (female, 36y, apparently healthy); and two reprogrammed at the D’Or Institute for Research and Education, CF1 (male, 37y, apparently healthy) and CF2 (male, 31y, apparently healthy) [13]. Organoids were additionally derived from human embryonic stem cell lines BR1 (Laboratory for Embryonic Stem Cell Research (LaNCE), University of São Paulo) [14] and H9 (RRID:CVCL_9773). Further information regarding those cell lines can be found in Additional file 1: Table S1. iPSCs and ESCs were cultured in mTeSR1 (Stemcell Technologies) or E8 medium (Thermo Scientific), on a Matrigel (BD Biosciences)-coated surface. Passaging of colonies was performed manually at 70% confluence and maintained at 37 °C in humidified air with 5% CO_2_. The establishment of hiPSCs and the derivation of NPCs, neurons, and organoids were carried out following international standards and with the approval of the local research ethics council (CAAE: 32385314.9.0000.5249).

### Human neural progenitor cells and neuronal differentiation

To induce the differentiation of hiPSCs into neural progenitors, we used a previously described protocol [15]. In brief, the hiPSC cultures from six individuals (three control and three schizophrenia patients), at 70% confluence, were induced into a neural lineage in a defined adherent culture by retinoic acid and basic fibroblast growth factor (bFGF). After 18 days, neural tube-like structures can be collected and plated on dishes coated with 10 μg/mL of poly-L-ornithine and 2.5 μg/mL of laminin (Thermo Fisher Scientific), in N2B27 medium (DMEM-F12 with 1 × N2 and 1 × B27 supplements, 1% penicillin/streptomycin) supplemented with 25 ng/mL bFGF and 20 ng/mL EGF (Thermo Fisher Scientific). Human neural progenitor cells (hNPCs) migrate from the neural tube-like structures and were thereupon tested for the expression of neural markers before expansion. Cell expansion was done in N2B27 medium supplemented with EGF and bFGF, replaced every other day. hNPCs were expanded for no more than 5 passages. Characterization of this culture has been previously published [16].

For neuronal differentiation, hNPCs of each lineage were plated on poly-L-ornithine- and laminin-coated dishes. To trigger differentiation, 24 h after plating bFGF and EGF were removed from the N2B27 medium. Neuronal cultures were differentiated for 21 days in vitro (DIV). The whole medium was replaced every 5 days. Both hNPCs and neuronal cells were incubated at 37ºC and 5% CO_2_. After the period described, cells were collected for proteomics or immunocytochemistry.

### Differentiation into brain organoids

For differentiation of schizophrenia and control human iPSCs and ESCs, cells were cultured in mTeSR1 medium (Stemcell Technologies) on Matrigel (BD Biosciences)-coated cell culture dishes. Differentiation of pluripotent stem cells into cerebral organoids was based on a previously described protocol [17, 18]. In summary, hiPSCs were passaged into single cells with Accutase (Merck Millipore) and inoculated in a spinner flask containing mTeSR1 medium supplemented with 10 μM Y-27632 (Rho-associated protein kinases inhibitor, iRock) (Merck Millipore) under constant rotation (40 rpm). The following day, the medium was replaced to initiate embryoid body formation. One week later, neural induction medium [DMEM/F12 1:1 supplemented with N2 (1x) supplement, 2 mM glutamax, 1% MEM-NEAA (Thermo Scientific), and heparin (1 μg/mL, Sigma)] was added. After 4 days, cellular aggregates were covered in Matrigel and cultured in differentiation medium [DMEM/F12:Neurobasal (1:1), supplemented with N2 (0.5x) and B27 minus vitamin A (1x) supplements, 2 mM glutamax, 0.5% MEM-NEAA, 0.2 μM 2-mercaptoethanol (Thermo Scientific), and 2.5 μg/mL insulin (Sigma)] for another 4 additional days. Subsequently, the medium was replaced with a neuronal differentiation medium, which comprises the same formulation, except for the use of 1 × B27 containing vitamin A (Thermo Scientific). This final medium was then changed every week for the differentiation of cerebral organoids, grown for 45 days, and collected for analyses. At 45-days, organoids present a well-organized structure, with both radial glia and a neuronal network [11, 17, 19], which is of interest in modeling initial developmental cues in schizophrenia.

### Immunohistochemistry

Cells were grown on coverslips for 5 days (for NPCs) or directly differentiated into neurons (for 21 DIV), washed with PBS to remove the medium, and fixed in 4% paraformaldehyde (PFA). Cerebral organoids were collected after 45 days of differentiation and immediately fixed in 4% PFA, followed by incubation in sucrose solutions over an increasing gradient (10, 20, and 30%) in phosphate-buffered saline (PBS). Subsequently, the organoids were embedded in an optimal cutting temperature compound (OCT) and frozen in liquid nitrogen. The organoids were sectioned with a cryostat (Leica) into 20 μm-thick sections. After being fixed, cells and organoids were washed with PBS, permeabilized in a 0.3% Triton-X solution, and blocked in a 3% bovine serum albumin (BSA) solution before immunolabeling. Immunofluorescence was performed using the primary antibodies: anti-MAP2 (Sigma-Aldrich Cat# M1406, RRID:AB_477171), anti-class III β-tubulin (Millipore Cat# MAB1637, RRID:AB_2210524), anti-nestin (Millipore Cat# MAB5326, RRID:AB_2251134), anti-PAX6 (Santa Cruz Biotechnology Cat# sc-11357, RRID:AB_2159706), anti-SOX1 (Millipore Cat# AB15766, RRID:AB_870981), and anti-SOX2 (Millipore Cat# AB5603, RRID:AB_2286686). Secondary antibodies used were AlexaFluor 488 goat anti-rabbit (Molecular Probes Cat# A-11008, RRID:AB_143165), AlexaFluor 488 goat anti-mouse (Thermo Fisher Scientific Cat# A-11001, RRID:AB_2534069), and AlexaFluor 594 goat anti-mouse (Molecular Probes Cat# A-11032, RRID:AB_2534091). DAPI was used for nucleus staining. Images were acquired using a Leica TCS SP8 confocal microscope.

### Sample preparation and processing

From each schizophrenia patient or control cell line, three independent NPCs (5 DIV) and neurons (21 DIV) samples, following protocols described earlier, were harvested, and pelleted in PBS. Similarly, five to six cerebral organoids were pooled from the spinner flask and pelleted in PBS to provide population variability within each patient or cell line. At least two batches of spinners were polled per cell line. Cell lines were then independently sampled and analyzed. Pellets of cells or organoids were removed from PBS and homogenized in lysis buffer [7 M urea, 2 M thiourea, 1% CHAPS, 70 mM DTT, and EDTA-free complete protease inhibitor cocktail (Roche)]. Sample lysates were kept on ice for approximately 20 min and centrifuged at 10,000 × *g* for 10 min at 4 °C; supernatants were collected, and protein content was quantified using a Qubit® 3.0 Fluorometer (Thermo Fisher Scientific). Each sample (100 µg) was subjected to a short SDS-PAGE run and a sequence of reduction, alkylation, and overnight trypsin digestion (1:50 w/w trypsin:total protein) at 37 °C. Peptides collected from this digestion were dried in a SpeedVac (Thermo Fisher Scientific) and stored at −80 °C until quantitative and qualitative shotgun mass spectrometry analyses.

### Liquid chromatography-mass spectrometry

Peptides were separated by a two-dimensional nanoAcquity UPLC M-Class System (Waters Corporation, Milford, MA) liquid chromatographer coupled to a Synapt G2-Si mass spectrometer (Waters Corporation). Organoid samples were separated by two-dimension chromatography, for the first-dimension reverse-phase chromatography, peptides (5 µg) were loaded onto an M-Class BEH C18 Column (130 Å, 5 µm, 300 µm X 50 mm, Waters Corporation). Peptide fractionation was performed by increasing steps of acetonitrile concentration (13%, 18%, and 50% ACN). Peptide loads were directed to a second-dimensional separation on an HSS T3 Column (100 Å, 1.8 µm, 75 µm X 150 mm, Waters Corporation, Milford, MA), with a binary gradient of 7% to 40% ACN (v/v) over 54 min at a flow rate of 0.4 µL/min. The fractionation for NPC and 21 DIV neuronal samples was performed in one dimension. Peptide loads were directed to separation on an HSS T3 Column (100 Å, 1.8 µm, 75 µm × 150 mm, Waters Corporation, Milford, MA), with a binary gradient of 7% to 40% ACN (v/v) over 54 min at a flow rate of 0.4 µL/min. In all cases, peptides entered the mass spectrometer using nano-electrospray ionization in positive ion mode, nanoESI ( +). The MS analyses were performed using data-independent acquisition (DIA) enhanced with ion mobility separation (HDMS^E^). [Glu1]-Fibrinopeptide B human was used as the lock mass compound, which was sampled every 30 s. Each cell line was considered a biological sample, and at least duplicates (for organoids) or triplicates (for NPCs and 21-DIV neurons) were run. The LC–MS/MS method used was based on a previously described protocol [20].

### Database search and quantitation

Raw data were aligned and processed in Progenesis® QI for proteomics version 3.0 (Waters). Protein identification and quantification were performed using the ion accounting algorithm with default parameters and searching against the *Homo sapiens* database—revised (Uniprot, version 2017/10). For protein identification, the following parameters were set: up to two missed cleavages for trypsin digestion; variable modification by oxidation (M) and fixed modification by carbamidomethyl (C), and False Discovery Rate (FDR) less than 1%. The quantitative analysis was carried out on the log2-values of the intensities after normalization by a software-calculated global scaling factor, and only proteins present in at least two out of three biological samples of both control and schizophrenia samples were considered as discovered. All proteins with an ANOVA p-value < 0.05, between the schizophrenia and control samples, were considered differentially expressed and selected for further analysis (Additional file 2: Table S2).

### In silico analysis

Gene ontology and pathway enrichment were analyzed with DAVID (the Database for Annotation, Visualization and Integrated Discovery) [21, 22] using whole-genome background; and Metascape [23], using default settings (whole proteome) and the following databases: KEGG Pathway, GO Biological Processes, Reactome and CORUM. Protein networks and canonical pathways associated with differentially expressed proteins were identified using Ingenuity Pathway Analysis software (IPA, Ingenuity Systems, Qiagen, Redwood, CA, USA; www.ingenuity.com). The significance of biological functions was calculated using Fisher's exact test. Multiple correlation hypotheses were calculated with the Benjamini-Hochberg (B-H) approach using a 1% FDR threshold; the significance levels of the IPA tests were expressed as p-values. Differentially regulated proteins in schizophrenia *postmortem* brain tissue were selected from the literature by searching the Pubmed database (https://pubmed.ncbi.nlm.nih.gov) using the following keywords: proteomics AND schizophrenia AND postmortem brain. Studies included were those with a list containing differentially regulated proteins, from several brain areas, such as the corpus callosum, anterior temporal lobe, anterior cingulate cortex, dorsolateral prefrontal cortex, mediodorsal thalamus, insular cortex, frontal cortex, Wernicke’s area, cerebellum, posterior cingulate cortex, caudate nucleus, and the hippocampus. The whole set of proteomic datasets was then compiled and is detailed in Additional file 3: Table S3. The compiled list of regulated proteins in *postmortem* brain areas of schizophrenia patients was then compared to the regulated proteomic data of PSC-derived schizophrenia versus control cerebral organoids, neurons, and NPCs. Comparative analyses were then performed in the Python programming language (v. 3.7.3). The overlap between proteins identified in our data and previous *postmortem* brain studies was visualized with a Chord diagram [24]. The over-representation analysis of brain regions and KEGG pathways was carried out using ClusterProfiler [25] in the R programming environment (v. 4.0).

## Results

**Fig. 1:**
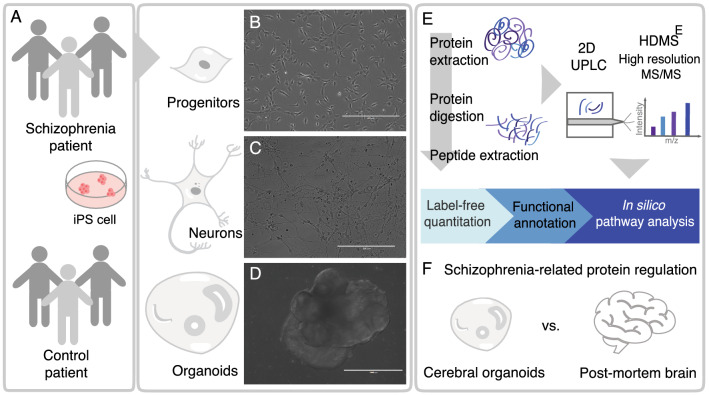
Schematic organization of the experimental design and analysis workflow. **A** Human iPSCs from schizophrenia patients and controls were differentiated into neural cell types: progenitors, neurons, and organoids. Representative photomicrographs of **B** neural progenitor cells (NPC), **C** neurons at 21 days in vitro*,* and **D** 45-day cerebral organoids. **E** Proteomics workflow processing. Label-free sample preparation (protein extraction and peptide digestion) followed by 2D-UPLC fractionation and on-line detection using HDMSE high-resolution MS/MS acquisition. Peptides and proteins were identified and quantified before functional annotation and other in silico analyses. **F** Differentially regulated proteins in cerebral organoids of schizophrenia patients were compared to available proteomics data from post-mortem brains of schizophrenia patients. Scale bars shown are **B** 400 µm, **C** 200 µm and **D** 1000 µm

Neurodevelopmental aspects of schizophrenia were investigated with the proteomic profiles of three models of neural differentiation: neural progenitors, immature neurons, and brain organoids. hiPSC from three schizophrenia patients and three paired controls were differentiated into neural cells and submitted to proteomic analyses, in a primary study to undercover dysregulated proteins, as presented in the workflow (Fig. 1). Each of the hiPSC samples from patients and controls (Fig. 1A) was differentiated into neural progenitor cells (NPCs) (Fig. 1B), young neurons with 21 days in vitro (21 DIV) (Fig. 1C), and brain organoids (cultured for 45 days) (Fig. 1D). Whole-cell proteomic profiles were generated using label-free quantitative proteomics (Fig. 1E) to uncover some of the molecular mechanisms of schizophrenia during neurodevelopment.

In addition, the proteomic data from organoids, 21 DIV neurons, and NPCs data (schizophrenia compared to controls, p < 0.05, as in Additional file 2: Table S2) were later further compared to a compiled list of publicly available proteomics datasets (Additional file 3: Table S3) from *postmortem* brain tissue of schizophrenia patients (Fig. 1F).

### *Establishing *in vitro* models of schizophrenia*

**Fig. 2:**
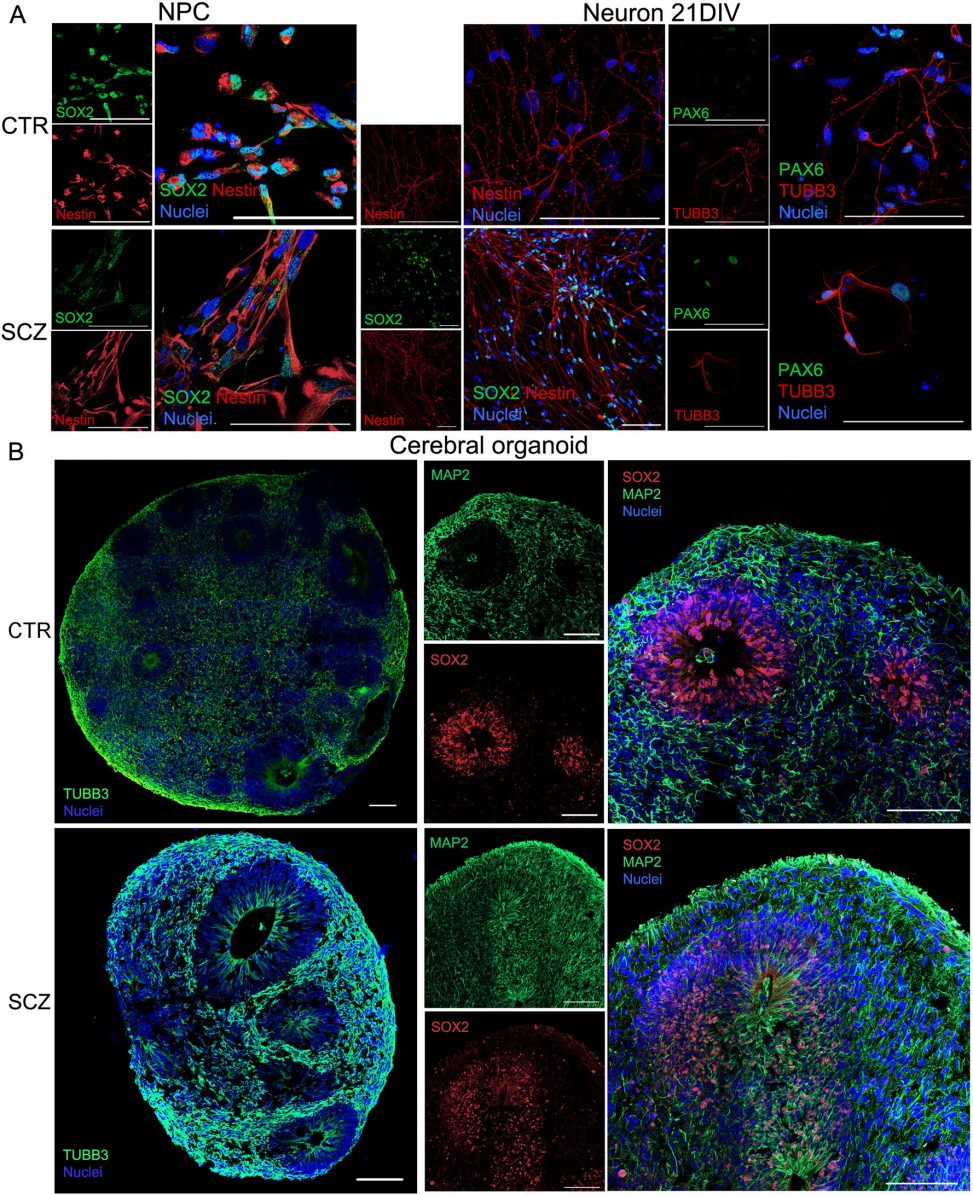
Morphological characteristics between controls and schizophrenia patients are similar in progenitors (NPCs), neurons (21 DIV), and cerebral organoids. **A** Immunocytochemistry characterization of NPCs and 21 DIV neurons. Representative micrographs of control NPCs and neurons in upper panels (CTR), and schizophrenia NPCs and neurons in the lower panels (SCZ). Showing SOX2 and nestin; PAX6 and TUBB3. Scale bars = 100 µm. **B** Immunocytochemistry of controls and SCZ-derived cerebral organoids showing ventricle-like morphology and SOX2 and MAP2 staining, as well as TUBB3. Scale bars = 100 µm

We first established cultures with progenitors and neurons differentiated from hiPSC cells of schizophrenia patients and controls, using a methodology we have previously characterized to provide mainly forebrain neural progenitors [26]. While NPCs were kept under a proliferative medium, neuronal differentiation was performed by removing FGF and EGF from the growth medium, switching the proliferation program to a neuronal one. At the end of each culture period, NPCs in both control and schizophrenia-derived cells stained positive for neural progenitor markers such as nestin and SOX2 (SRY-Box 2) (Fig. 2A). Differentiation of progenitors into young neurons was also induced in both schizophrenia and control cells. After 21 days of differentiation in vitro (21 DIV), morphological changes were observed in both control and schizophrenia cells, showing small cell bodies and a large number of cells with neurite elongation (Fig. 2A). Neurons at 21 DIV continued to stain for nestin and SOX2, with few cells staining for PAX6 (paired box 6), while several stained for β3-tubulin (TUBB3) (Fig. 2A), and fewer showed matured MAP2 staining (microtubule-associated protein 2) (Additional file 4: Figure S1A). Proteomic analysis also indicated the expression of nestin and TUBB3; in progenitors, the transcription factor PAX6 was observed, though it was not detected in neurons (Additional file 2: Table S2). Gene expression analyses (Additional file 4: Figure S1B) have shown that 21 DIV neurons express neuronal associated cytoskeleton, such as MAP2 and TUBB3, and synapses-related mRNA expression such as PSD-95 (postsynaptic density 95) and increased synaptophysin (SYP) in comparison to progenitors. In addition, as the differentiation protocol was not directed to particular brain regions, neuronal differentiation is heterogeneous. We observed the expression of genes related to the GABAergic systems, as shown by glutamic acid decarboxylase (GAD65), as well as genes involved in the differentiation of midbrain dopaminergic neurons, such as NURR1 (nuclear receptor-related 1 protein, or NR4A2) and TH (tyrosine hydroxylase), to be increasing in 21 DIV neurons in comparison to progenitors (Additional file 4: Figure S1B). Instead, the glutamatergic system, such as glutaminase (GLS), glutamate transporter (VGLUT1), and NMDA receptor (NMDAR), are present yet more prominent in progenitors. Glutamate is responsible for regulating neurogenesis, and its activity in progenitors influences neurodevelopment, secreting other neurotrophic factors. Our results point to a neuronal diversity toward increasing dopaminergic and GABAergic developmental cues in the differentiation protocol. In the human fetal cortex, NMDAR regulation in progenitors might directly increase the number of TH-positive neurons, regulating cell proliferation and the production of dopaminergic cells [27, 28].

To evaluate a more complex model of cellular organization in vitro, we generated cerebral organoids at 45 days, a point at which a neuronal network has already been initially formed [11, 17]. The whole-cerebral organoid protocol used has no cues for inducing a specific region, and the self-organized cytoarchitecture in both controls and schizophrenia-derived organoids was similar. The organoids from control and schizophrenia showed no major morphological differences, and 45-day-old cerebral organoids developed one to several putative ventricles, with young neurons identified by TUBB3-positive cells found at the subventricular zone in both control- and schizophrenia-derived organoids (Fig. 2B). Control organoids showed organized ventricle zones with SOX2-expressing progenitor cells and MAP2-expressing neuronal cells at the cortical layer, while schizophrenia patients had a larger area covered by SOX2 progenitors, also showing MAP2 distribution throughout the organoid (Fig. 2C). In summary, these morphological aspects and protein markers confirm the models as suitable for comparison of schizophrenia and control differences during neurodevelopment.

### The proteomics of schizophrenia-derived neural cells

Proteomic analyses were performed at three different developmental models of hiPSC-derived neural cells: neural progenitor cells (NPCs), neurons at 21 days in vitro (21 DIV), and cerebral organoids at 45 days (Additional file 2: Table S2). The NPC dataset yielded a total of 1949 quantified proteins, of which 364 proteins were found to be dysregulated (p < 0.05) between schizophrenia-patients NPCs and controls. Of those proteins, 84% (306) were downregulated (Additional file 2: Table S2). In the 21 DIV neuronal model, 1833 proteins were quantified, 264 of which were considered dysregulated (p < 0.05) between schizophrenia and controls. Of these proteins, 70% (185) were downregulated. A slightly larger number of proteins was quantified from the 45-day cerebral organoids, which yielded 2177 quantified proteins. Between schizophrenia and control groups, 535 proteins were considered dysregulated (p < 0.05), 59% (317) of which were downregulated.

**Fig. 3:**
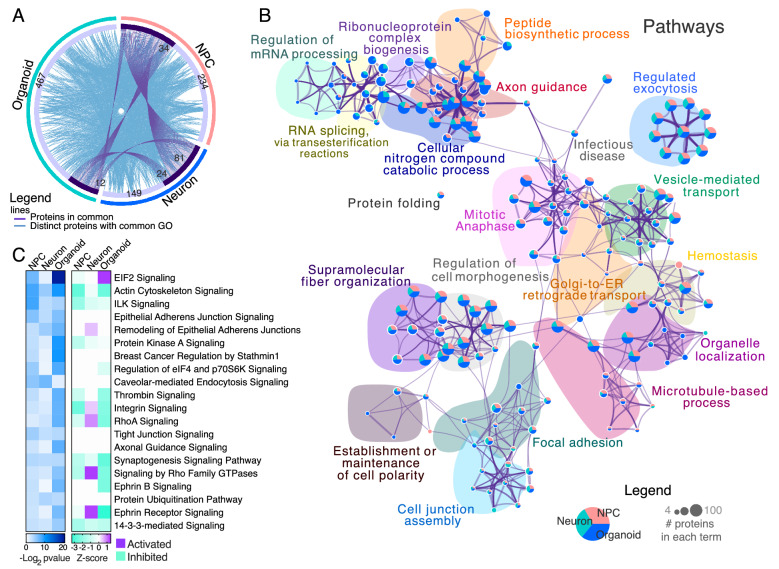
Differential regulation of proteins in schizophrenia patients versus control cells from human PSC-derived organoids, neurons, and NPCs. **A** Chord diagram overviewing individual proteins (purple lines) and pathways (blue lines) shared among comparisons. The numbers of proteins (total and overlapped) are detailed. **B** Functional network tree representation of enriched pathways, processes, and GO terms. Terms were grouped by similarity metric (kappa scores) and the most statistically significant term within a cluster was set to represent the cluster, according to Metascape analyses. **C** Canonical pathway analyses were performed using the Ingenuity pathway analyses dataset. The blue plot represents pathway enrichment (Log2 adjusted p-value), and the purple/green plot represents z-scores prediction of activation/inhibition of those pathways. Purple represents activated scores, and green represent inhibited scores

When comparing dysregulated proteins of schizophrenia versus control between the three cellular models, we found that some dysregulated proteins were common among NPC, neurons, and cerebral organoids (Fig. 3A, represented by purple lines), which points to several disrupted pathways at all developmental models (Fig. 3A, represented by blue lines). Twelve dysregulated proteins were commonly found in all models comparing schizophrenia with controls, most of them downregulated in the three models, which included i.e., 14–3-3 protein eta (YWHAH) and spectrin beta chain (SPTBN2); and others, such as poly [ADP-ribose] polymerase 1 (PARP1), were shown upregulated in organoids (Additional file 4: Figure S2).

We have selected high abundant proteins with varied unique peptides from the dysregulated list (YWHAH, MAP4, THY1, and CTNNB1), independently of being up- or downregulated, for validation by a multiple reaction monitoring method. Both analyses (shotgun and MRM) are different, and protein quantification can be distinct due to specific peptides chosen for intensity quantification, and post-translational modifications content (which were not included in these searches). Therefore, we have validated the presence of the protein by MRM qualitatively, yet not all were as well quantitatively equal (Additional file 4: Table S4).

Combining these observations of dysregulated proteins in schizophrenia (versus control), constituent pathways can be observed in more detail (Fig. 3B). Organoids and neurons have deregulation in mRNA processing, including splicing. This is followed by disruptions in protein synthesis and folding at all cell models analyzed, which are presumed to have downstream consequences on axon guidance, exocytosis, cell–cell adhesion, and cytoskeleton organization, disrupting hemostasis, and cell maintenance.

Ephrin B and ephrin receptor signaling canonical pathways were dysregulated in schizophrenia-derived cerebral organoids, NPCs, and neurons in comparison with controls. Our data indicate a major downregulation of proteins involved in Ephrin signaling (-Log_10_ p-value– organoids: 6.53; NPCs: 2.60; neurons: 1.24) and further prediction analysis using Ingenuity Pathway Analysis offered some possible outcomes (Fig. 3C). Another family of proteins found enriched in all analyses was 14–3-3 signaling (-Log_10_ p-value– organoids: 4.96; NPCs: 2.97; neurons: 2.44), indicating inhibition of this pathway. Axon guidance (-Log_10_ p-value – organoids: 7.68; NPCs: 2.63; neurons: 1.25) and synaptogenesis (-Log_10_ p-value – organoids: 3.74; NPCs: 3.77; neurons: 3.54) were also found dysregulated in all schizophrenia neural cells (Fig. 5). All these pathways have been previously associated with schizophrenia [29], and they perform essential roles in brain development.

**Fig. 4:**
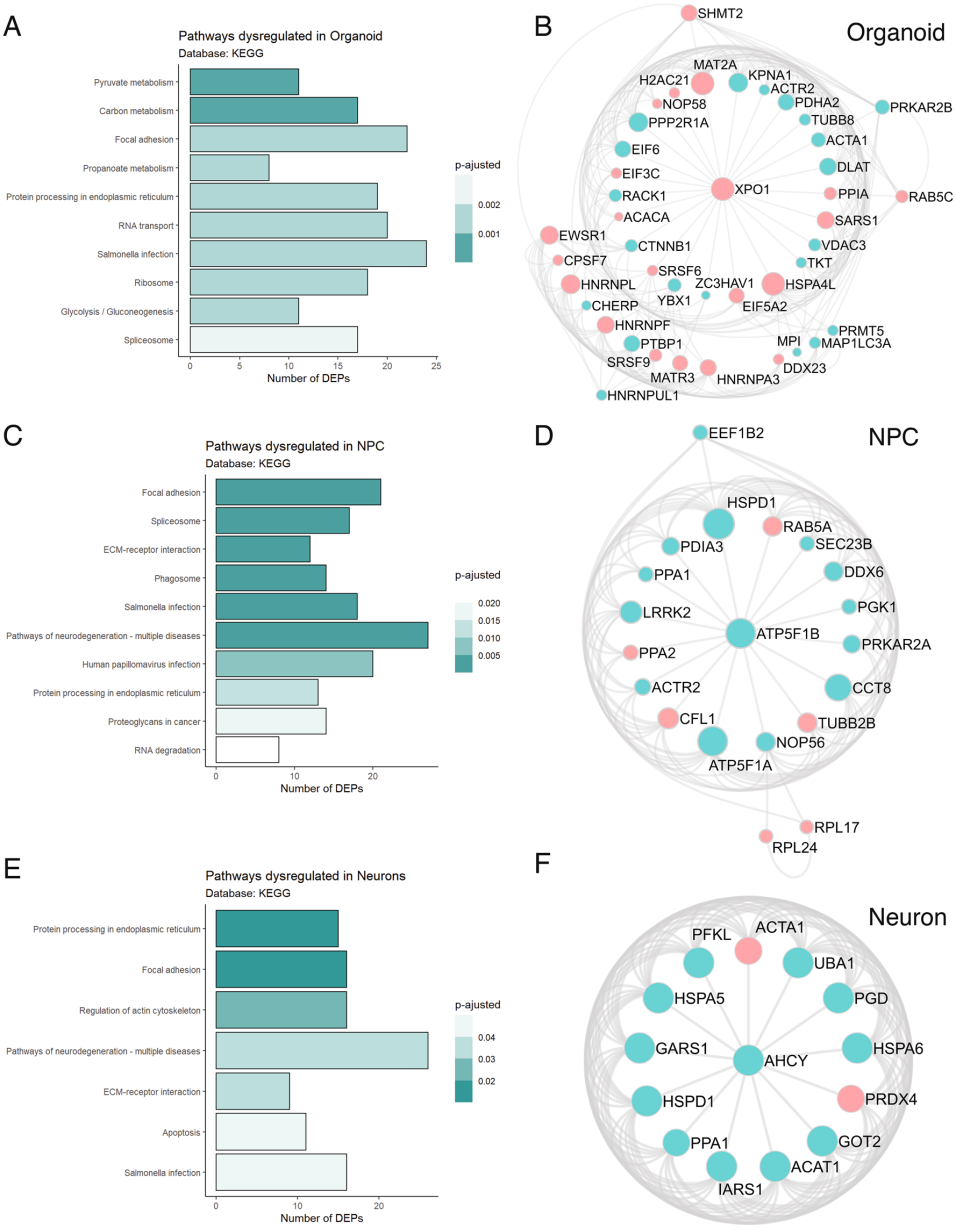
Pathway regulation and protein–protein interaction (PPI) visualization in organoids, neurons, and NPCs of schizophrenia patients versus controls regulated proteins. KEGG pathways found dysregulated by the differentially expressed proteins (DEP) from schizophrenia patients versus controls in **A** organoids, **C** NPCs, and **E** 21 DIV neurons are individually shown. Bars represent the number of DEPs found in a particular pathway and the color represents the enrichment adjusted p-value. The protein–protein interaction in **B** organoids connects proteins found regulated in mRNA splicing and metabolism of RNA. PPI of proteins regulated in **D** NPCs are related to translation, axon guidance, and oxidative phosphorylation. And PPI of proteins regulated in **F** neurons are part of cellular amino acid metabolic process and cytosolic tRNA aminoacylation. Circles in red indicate upregulation of proteins and green indicate downregulation of proteins. Circle size reflects the interaction degree of each protein. Analyses were performed using Metascape resources

Individual analyses of organoids, NPCs, and neurons derived from patients with schizophrenia are represented in Fig. 4 (A, C, and E). Furthermore, the protein–protein interaction (PPI) analyses performed by Metascape show that schizophrenia-derived cerebral organoids presented dysregulated proteins that are involved in mRNA splicing and RNA metabolism (Fig. 4B). Schizophrenia-derived NPCs presented proteins related to translation and oxidative phosphorylation (Fig. 4D). Lastly, proteins seen in schizophrenia-derived neurons (21 DIV) are associated with amino acid metabolic processes and cytosolic tRNA aminoacylation (Fig. 4F).

### Similarities among hiPSC-derived neural cells, brain organoids, and schizophrenia postmortem brains

**Fig. 5:**
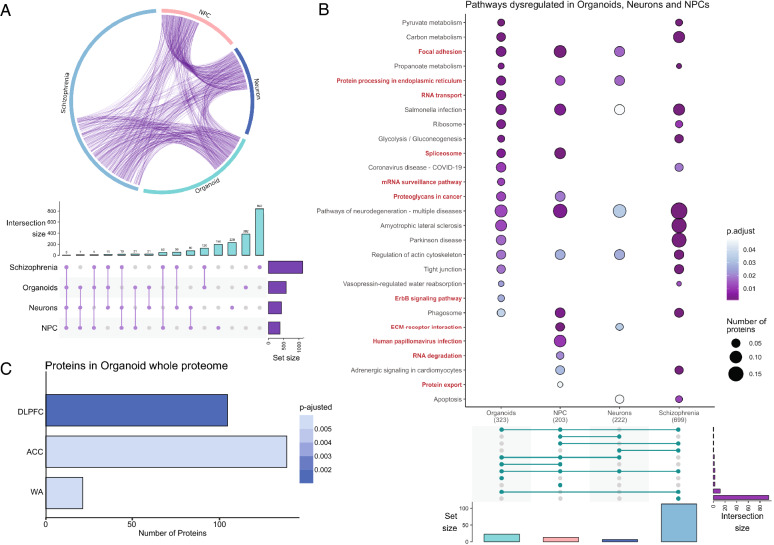
Proteomic from iPSC-derived neural cells and organoids from schizophrenia patients present similar patterns when compared with post-mortem brain tissue of patients with schizophrenia. **A** Chord-diagram (upper) overviewing proteins (purple lines) shared among comparisons; upset plot (lower) indicating the number of proteins shared for each comparison. Vertical bar sizes (intersection size) represent the number of proteins in common among the set groups, where the purple dots (and connecting line) are pointing to where this intersection (or overlap) occurs; and the horizontal bars represent the number of proteins differentially regulated (SCZ versus CTR) in each dataset (set size). **B** Canonical pathway enrichment analysis against the KEGG database (upper), showing pathways regulated by protein present in each dataset, followed by an upset plot indicating the connections of those pathways among organoids, 21 DIV neurons, NPCs, and the *postmortem* brain. Highlighted terms (bold red) represent novel pathways found in this study, not covered in *postmortem* brain tissues. Color shades (purple to blue) are representative of the p-value, while circle sizes represent the number of proteins within respective canonical pathway. Vertical bars represent the number of pathways regulated in each dataset (set size); horizontal bars (intersection size) represent the number of pathways in common among the set group; green dots (and connecting lines) are pointing to where this intersection occurs. **C** Comparative analysis of total organoids dataset with proteins from different regions of the *postmortem* brain. Bars represent the number of proteins, while they were colored according to their adjusted p-value of the analyses overlap. *DLPFC* dorsolateral prefrontal cortex, *WA* Wernicke’s area, *ACC* nterior cingulate cortex

Several of the results obtained with hiPSC-derived neural cells and organoids were similar to those previously described in studies with *postmortem* brain tissue from schizophrenia patients. Observing such similarities, we questioned whether organoids derived from patient cells could be used as an in vitro model to study aspects of the pathophysiology of schizophrenia. Thus, we compiled a list of proteomics data containing several of the available studies in *postmortem* brain proteomics from the literature considering only the dysregulated proteins (in comparison to healthy individuals) of several brain regions (Additional file 3: Table S3). Then, we have compared that dysregulated list to the hiPSC-derived neural cells and organoids data from schizophrenia patients versus healthy controls of this work (p < 0.05, Additional file 2: Table S2). The signaling pathways and proteins that were found in common between hiPSC-derived neural cells/organoids and brain tissue are shown in Fig. 5. A total of 126 proteins were found commonly regulated when comparing organoids and *postmortem* brain tissue list, while 50 were found commonly regulated between NPCs and brain tissue, and 56 between neurons and brain tissue (Fig. 5A).

There is a high intersection of proteins between organoids and *postmortem* brain analyses of patients with schizophrenia (Fig. 5A), which is followed by a similar outcome in terms of canonical pathways (Fig. 5B), with pathways such as focal adhesion, protein processing, and pathways on neurodegeneration, corresponding to some of the several representative pathways modified. ErbB signaling pathway, RNA degradation and protein export, are for instance canonical pathways not commonly overrepresented in the postmortem dataset. The intersection shows higher similarity between organoids and *postmortem* brain (in comparison to NPC and neurons), thus, we followed with a comparative analysis of brain regions. Using proteins identified in the organoids, we compared different *postmortem* brain regions' specific expression, as to identify which of those regions would have greater similarity to the organoid data. Comparing those proteins on the dataset and the organoids derived, we found the dorsolateral prefrontal cortex (DLPFC), followed by Wernicke’s area (WA) and the anterior cingulate cortex (ACC) (Fig. 5C), as those with the stronger correlation, and therefore similarity to the organoids.

## Discussion

Due to the difficulty in obtaining brain tissue from fetuses, along with ethical considerations, animal models were almost exclusively used to study and understand neurodevelopment. hiPSC cultures, especially 3D models such as organoids, hold great promise for the study of neurodevelopment and complex, multifactorial diseases since these cells preserve the genetic background of patients [30, 31]. Considering the cell microenvironment necessary for development, it is of particular interest to study patient-derived 3D brain organoids in the disease context. And it is important to state that those are experimental cell models, which although not representing the actual human brain, can provide valuable data to understand the early human neurodevelopmental paths disrupted in disease.

The neurodevelopmental hypothesis of schizophrenia is largely accepted and is based on structural, cognitive, and motor abnormalities that are identified in childhood and coincide with the development of the disease as adults (reviewed in [32, 33]). Furthermore, environmental factors such as hypoxia, viral infections, perinatal injury, and maternal drug abuse have been considered major risk factors for the development of this disease [34–40]. It is for this reason that this study investigated the proteins expressed in brain organoids (45 days), neural progenitor cells, and immature neurons (21 DIV) to understand the neurodevelopmental machinery at work in the cells of patients with schizophrenia.

### Early dysregulation of axonal guidance and synaptogenesis signaling in schizophrenia at the protein level

Several of the main dysregulations observed here are related to the development of neurons and their connections, which will drive the formation of synapses in the brain in development. The ephrin receptor signaling pathway (Fig. 3C) is one of those found dysregulated in all the models of neural cells studied. Previous studies using genetic approaches, both in vivo and in vitro, have shown the role of this pathway in important processes of neural development such as axon guidance and dendritic spine formation [41–45]. This canonical pathway was also found to be dysregulated in the *postmortem* brains of patients with schizophrenia [46].

The decrease of constituent proteins of the ephrin pathway impairs the formation of dendritic spines [41, 47] and *postmortem* tissue studies show that patients with schizophrenia have a reduced number of dendritic spines [48]. Both in organoids and NPCs, the ephrin receptor signaling pathway was predicted to be inhibited (IPA canonical pathway analysis, Fig. 3C). In contrast, this pathway was predicted to be activated in neurons, suggesting that regulatory mechanisms are being triggered in this cell type to reverse the dysregulation in synaptic formation observed in neural progenitor cells (NPCs). The ephrin pathway is known to regulate NMDA receptor activity [47], which has already been widely associated with the pathophysiology of schizophrenia, and glutamatergic dysfunction is another hypothesis for the etiology of the disease [49]. NMDA hypofunction in neurodevelopment causes changes in the number of dendritic spines and the development of the neural circuit [50]. Recent GWAS studies [51] have pointed to several genes associated with neuronal function, which implicates modifications in cell differentiation, the transmission of synapsis, and synaptic organization being altered in patients with schizophrenia. Those associations were mostly found in genes expressed in both excitatory and inhibitory neurons. And, although pre- and post-synaptic proteins are not shown altered in our models, possibly due to the models being young and still immature, dysregulations, mostly downregulations occur in disease, and several proteins participate in pathways leading to future synapsis impairment.

Another signaling pathway found dysregulated in all three analyses was 14–3-3 (Fig. 2C). These proteins are abundant in the mammalian brain and comprise 1% of the cerebral proteome [52], playing roles in cell cycle regulation, apoptosis, differentiation, and migration, as well as more complex processes such as neurotransmission, neuroplasticity, and synaptogenesis (reviewed in [53]). Proteins of the 14–3-3 pathway have also been specifically associated with the pathophysiology of schizophrenia [46, 54]. Functional knockout of 14–3-3 family proteins lead to deficits in neuronal migration and structural organization of cells of the cortex and hippocampus [55, 56], as well as behavioral changes and cognitive deficits [57, 58]. The protein 14–3-3 eta (YWHAH), in particular, was found downregulated in NPCs, neurons, and organoids. It is a protein located in a chromosomal region (22q12), which is considered a high-risk region for developing schizophrenia; and RNA levels of this protein have been found downregulated in *postmortem* cerebellum [59–61]. In addition, this protein has been found altered in four distinct *postmortem* brain regions of patients with schizophrenia [62–64].

The actin cytoskeleton signaling pathway, which was found to be dysregulated in NPCs, neurons, and organoids, and predicted to be inhibited, plays a role in the movement of growth cones, maintenance of neural polarization, formation of dendritic spines, and formation/stabilization of the synapse, functions that are known to be affected in patients with schizophrenia (reviewed in [65]). The Rho GTPase family regulates actin in neuronal morphology [66]. In NPCs and organoids, RhoA signaling and Rho family GTPases pathway were predicted to be inhibited; and both transforming protein RhoA (RHOA) and actin-related protein 2 (ACRT2) were found downregulated in the schizophrenia-patients’ cells. However, in neurons, we have found those canonical pathways predicted to be more active, with Rho guanine nucleotide exchange factor 4 (ARHGEF4) and Rho GTPase-activating protein 45 (ARHGAP45) upregulated in these cells. As the Rho GTPase family plays a role in the regulation of both axon guidance and synaptogenesis (Additional file 4: Figure S3 and 4, respectively), the activation of these proteins in neurons is probably a cellular response to restore actin signaling and consequently proper synaptic formation, which is impaired in NPCs derivate of patients with schizophrenia [67]. Synaptic impairment was also observed in a transcriptome study of brain organoids [68], and similarly, a decrease of synaptic proteins and neural connection was observed in an in vivo proteomic study of neural progenitor cells from schizophrenia patients [4]. Overall, these results indicate a possible dysregulation in the formation and maturation of synapses during development, especially regarding axon guidance and synaptogenesis and it is in line with previous hiPSC and *postmortem* tissue studies.

### Organoids and immature neurons exhibit differences in main biochemical pathways and protein–protein interactions when compared with previous hiPSC studies

Spliceosome-associated proteins were enriched in patient-derived organoids. The splicing process is coordinated by five multi-megadalton ribonucleoproteins (snRNPs) forming a complex. Malfunctions in the splicing complex can lead to several diseases, many observed in the nervous system [69], mostly due to neurotransmission dysregulation, especially regarding the GABAergic, dopaminergic, and glutamatergic systems [70, 71]. Moreover, genes related to brain development and maturation demonstrate abnormal regulation of alternative splicing in schizophrenia [72].

Some proteins of the snRNP complex, the core component of the splicing operation, such as heterogeneous nuclear ribonucleoproteins L (HNRNPL), F (HNRNPF), and A2/B1 (HNRNPA2), along with heat shock 70 kDa protein 4L (HSPA4L), were found upregulated in the schizophrenia-derived organoids. These proteins have been previously found in schizophrenia studies involving *postmortem* brains and in vitro techniques [73–76]. No previous study, observing hiPSCs derived cells from schizophrenia patients had indicated dysregulation in this pathway, placing focus on this target for future hiPSC studies.

PPI interaction analyses revealed a main dysregulation in oxidative phosphorylation (OXPHOS) of patient-derived neural progenitor cells (NPCs). Defects in this pathway during neurodevelopment have been associated with several psychiatric diseases [77]. OXPHOS is especially important in neural development, a process that requires high energy demand, though it also supports signaling processes, calcium homeostasis, and reactive oxygen species (ROS) production (reviewed in [78]). Proteins of the OXPHOS complex have been found dysregulated in four *postmortem* brain regions of patients [79]. Previous studies have pointed to differential gene expression [68] related to mitochondria function and morphological changes [4] in mitochondria of hiPSC-derived NPCs from schizophrenia patients, which complemented by protein dysregulation, suggest functional impairment in this organelle in the disorder. Lastly, dysregulation of energy metabolism in schizophrenia has been observed in neuroimaging and *postmortem* brain studies [79, 80], including reduced OXPHOS activity in the prefrontal cortex of schizophrenia patients evaluated by integrated proteomics, metabolomics, and genomics [81].

On the other hand, the metabolism regulation of young neurons in schizophrenia patients, as found in the PPI network, falls under cellular amino acid metabolic processes and cytosolic tRNA aminoacylation. Several studies have shown that differences in the level of amino acids are associated with schizophrenia patients (reviewed in [82]). And this is the first time we are able to point those dysregulations on hiPSC-derived neurons in schizophrenia studies, indicating new targets for future in vitro neuronal developmental studies using iPSC. Amino acids, such as glycine and serine, play an important role in the neurotransmission system. In the context of NMDA receptor activation, both glycine and D-serine have the same function; however, it has been hypothesized that in neurodevelopment, D-serine may have greater importance than glycine, since it is found in abundance during this period, as well as being co-located with NMDA receptors [50]. Defects in glycine binding to its receptor have been linked to cognitive and negative symptoms of schizophrenia [83], while a decrease in D-serine has been observed in different brain regions, the blood, and the cerebral spinal fluid of schizophrenia patients (reviewed in [84]). Knockout mice for serine racemase are also used as an established model of schizophrenia because they satisfactorily mimic NMDA receptor hypofunction [85].

### The prefrontal cortex stands out in schizophrenia development when comparing brain organoids and regions of postmortem tissue

The dorsolateral prefrontal cortex (DLPFC) is located in the prefrontal cortex and is involved in working memory and attention [86], both of which are known to be altered in patients with schizophrenia [87–90]. There is an excess of synapse pruning in the DLPFC that can reach up to 30% more than what occurs in a healthy individual (reviewed in [88]). This loss of synaptic connections in patients with schizophrenia usually occurs in late adolescence or early adulthood, a period marking the end of neurodevelopment and the point at which the onset of the disease usually occurs. In addition, this increase in synaptic pruning is related to the previous appearance of prodromal symptoms; that is, this dysregulation begins before the noticeable onset of the disease and is linked to synaptic formation [88].

Gulsuner et al. [91] mapped de novo mutations in fetal DLPFC, taking into consideration those who were later diagnosed with schizophrenia; genes for proteins that play a role in axon guidance, synaptic transmission, and transcriptional regulation were all highlighted. These results are consistent with those obtained in this study, reinforcing the role of the synaptic system, especially in the prefrontal cortex, in brain development and the emergence of schizophrenia from a pathophysiological point of view.

The results presented here have shown that several essential pathways for neurodevelopment, such as axon guidance and synaptogenesis, have constituent proteins with reduced abundance in schizophrenia. Similar neurodevelopmental aspects are observed in the three patients, and despite their distinct genetic and symptomatic backgrounds, differential proteins were consistently observed. The strength of our study using neural-derived models of hiPSCs from patients with schizophrenia is a pronounced 89% of dysregulated proteins found to be downregulated, which we hypothesize to be a delay or slowing in brain development, thus reinforcing the hypothesis of schizophrenia as a neurodevelopmental disease. Our findings, as a developmental model of the disease, reinforce several of the aspects studied in schizophrenia, as shown by the similarities to the dysregulation found in *postmortem* brain studies, and could be further confirmed with a larger dataset, as to specific focus on the pathways presented here. Those similarities indicate that distinct genetic aspects converge to similar and common dysregulation in schizophrenia, which we can already observe in initial differentiation, leading to disruptions in neurodevelopment common to the disease. In addition to causing problems during neurodevelopment, these deregulations persist in the adult individual and thus contribute to the multifactorial nature of the pathophysiology of schizophrenia. Much of the data found in these analyses agree with previous studies using hiPSC cells from patients with schizophrenia, both confirming the data from this study and reinforcing the findings of the previous experiments. Nonetheless, new overlaps were also found in amino acid metabolism and spliceosomes, indicating new targets of study, at both the level of individual proteins as well as the level of biological pathways to which these proteins belong. Despite having a reduced number of subjects whose cells were hiPSCs sources, these data confirm the viability of *postmortem* tissue in proteomic studies, and further support patient-derived cell cultures as a valid technique to study schizophrenia.

## Supplementary Information

**Additional file 1: Table S1.** Cell lines information

**Additional file 2: Table S2.** Protein expression of iPSC-derived NPCs, neurons (21 DIV), and cerebral organoids from schizophrenia patients.

**Additional file 3: Table S3.** Compilation of proteomic studies in different regions of the postmortem brain.

**Additional file 4.** Additional tables and figures.

## Data Availability

The proteomic datasets generated for this study can be found in the PRIDE proteomics data repository (https://www.ebi.ac.uk/pride/archive/) with the accession numbers PXD026381 and PXD026593.
