# Supplementary material for: Proteomic signatures of schizophrenia-sourced iPSC-derived neural cells and brain organoids are similar to patients' postmortem brains
Source: Cell Biosci. 2022 Dec 1;12:189. doi: 10.1186/s13578-022-00928-x (PMC9714120; doi:10.1186/s13578-022-00928-x)
Supplement: Supplementary file 4 — Additional file 4. Additional tables and figures. [file 13578_2022_928_MOESM4_ESM.pdf]

Proteomic signatures of schizophrenia-sourced iPSC-derived neural cells and brain organoids are similar to patients' postmortem brains

Juliana M. Nascimento[1,2,#], Verônica M. Saia-Cereda[1,#], Giuliana S. Zuccoli[1], Guilherme Reis-de-Oliveira[1], Victor Corasolla Carregari[1], Bradley J. Smith[1], Stevens K. Rehen[2,3*], Daniel Martins-de-Souza[1,2,4,5*]

Additional file content

Supplementary Methods.

Table S4-S6

Figures S1-S4

Supplementary Methods

Multiple Reaction Monitoring (MRM) for targeted protein.

Approximately 1 µg of digested peptides from cell cultures and organoids (all cell types and differentiations) were loaded onto a Symmetry C18 5 µm, 180 µm × 20 mm precolumn (Waters Corporation, Milford, MA, USA) used as trapping column and subsequently separated by a 60 min reversed phase gradient at 300 nL/min (linear gradient, 3–50% ACN over 60 min) using a HSS T3 C18 1.8 µm, 75 µm × 150 mm nanoscale and LC column (Waters Corporation) maintained at 40 °C. For the gradient elution water-Formic Acid (99.9/0.1, v/v) has been used as eluent A and Acetonitrile Formic Acid (99.9/0.1, v/v) as eluent B. The separated peptides have been analysed by High Definition Synapt G2-Si Mass spectrometer directly coupled to the chromatographic system.

The generated raw file was imported in the Skyline software and the targeted proteins were chosen as a FASTA file to be used as a reference for the theorical tryptic peptides, isolating the mass of the peptides of interest (parent ions) used for the MS/MS selection (fragment ions) and their retention time to create the MRM method. The selected proteins to construct the MRM method were Q04917, P27816, P04216, Q00839, P35222 and P32004.

All the transitions choose for each MRM method is listed in the table below:

Table S4: List of Multiple Reaction Monitoring (MRM) transitions

Q04917 - 1433F_HUMAN 14-3-3

- K.AVTELNEPLSNEDR.N (793.8865++)
  - precursor - 793.8865++
  - precursor [M+1] - 794.3879++
  - precursor [M+2] - 794.8892++
  - N [y9] - 1073.4858+
  - E [y8] - 959.4429+
  - P [y7] - 830.4003+
- R.NLLSVAYK.N (454.2660++)
  - precursor - 454.2660++
  - precursor [M+1] - 454.7675++
  - precursor [M+2] - 455.2688++
  - L [y6] - 680.3978+
  - S [y5] - 567.3137+
  - V [y4] - 480.2817+
- K.NSVVEASEAAYK.E (634.3119++)
  - precursor - 634.3119++
  - precursor [M+1] - 634.8133++
  - precursor [M+2] - 635.3147++
  - E [y8] - 868.4047+
  - A [y7] - 739.3621+
  - S [y6] - 668.3250+
- K.DSTLIMQLLR.D (595.3341++)
  - precursor - 595.3341++
  - precursor [M+1] - 595.8355++
  - precursor [M+2] - 596.3361++
  - L [y7] - 886.5543+
  - I [y6] - 773.4702+
  - M [y5] - 660.3861+

P27816 – MAP4_HUMAN

- K.TTDMAPSK.E (425.7024++)
  - precursor - 425.7024++
  - precursor [M+1] - 426.2038++
  - precursor [M+2] - 426.7038++
  - T [y7] - 749.3498+
  - D [y6] - 648.3021+
  - M [y5] - 533.2752+
  - P [y3] - 331.1976+
- K.DMELPTEK.E (481.7286++)
  - precursor - 481.7286++
  - precursor [M+1] - 482.2301++
  - precursor [M+2] - 482.7303++
  - M [y7] - 847.4230+
  - E [y6] - 716.3825+
  - L [y5] - 587.3399+
  - P [y4] - 474.2558+
- K.ALPLEAEVAPVK.D (618.8635++)
  - precursor - 618.8635++
  - precursor [M+1] - 619.3650++
  - precursor [M+2] - 619.8664++
  - P [y10] - 1052.5986+
  - E [y8] - 842.4618+
  - A [y7] - 713.4192+
  - E [y6] - 642.3821+
  - P [y3] - 343.2340+
- K.DMAQLPETEIAPAK.D (757.3820++)
  - precursor - 757.3820++
  - precursor [M+1] - 757.8834++
  - precursor [M+2] - 758.3843++
  - L [y10] - 1068.5936+
  - P [y9] - 955.5095+
  - E [y8] - 858.4567+
  - P [y3] - 315.2027+
- K.ATPMPSRPSTTPFIDK.K (873.4482++)
  - precursor - 873.4482++
  - precursor [M+1] - 873.9496++
  - precursor [M+2] - 874.4506++
  - P [y12] - 1345.7110+
  - R [y10] - 1161.6262+
  - P [y9] - 1005.5251+
  - S [y8] - 908.4724+
  - P [y5] - 619.3450+

P04216- THY1_HUMAN

- R.HENTSSSPIQYEFSLTR.E (998.4740++)
  - precursor - 998.4740++
  - precursor [M+1] - 998.9754++
  - precursor [M+2] - 999.4768++
  - P [y10] - 1253.6525+
  - I [y9] - 1156.5997+
  - Q [y8] - 1043.5156+
- K.VLYLSAFTSK.D (564.8186++)
  - precursor - 564.8186++
  - precursor [M+1] - 565.3201++
  - precursor [M+2] - 565.8215++
  - Y [y8] - 916.4775+
  - L [y7] - 753.4141+
  - S [y6] - 640.3301+

P35222- CTNB1_HUMAN

- R.TMQNTNDVETAR.C (690.3146++)
  - precursor - 690.3146++
  - precursor [M+1] - 690.8160++
  - precursor [M+2] - 691.3166++
  - T [y8] - 905.4323+
  - N [y7] - 804.3846+
  - D [y6] - 690.3417+
- R.CTAGTLHNLSHHR.E (752.3653++)
  - precursor - 752.3653++
  - precursor [M+1] - 752.8666++
  - precursor [M+2] - 753.3674++
  - L [y8] - 1013.5388+
  - H [y7] - 900.4547+
  - N [y6] - 763.3958+
- R.MEEIVEGCTGALHILAR.D (949.9768++)
  - precursor - 949.9768++
  - precursor [M+1] - 950.4782++
  - precursor [M+2] - 950.9789++
  - G [y11] - 1168.6255+
  - C [y10] - 1111.6041+
  - T [y9] - 951.5734+
- R.MSEDKPQDYK.K (620.7794++)
  - precursor - 620.7794++
  - precursor [M+1] - 621.2808++
  - precursor [M+2] - 621.7814++
  - D [y7] - 893.4363+
  - K [y6] - 778.4094+
  - P [y5] - 650.3144+

Quantitative Real Time Quantitative PCR (qRT-PCR)

For the characterization of neuronal markers, total RNA of control progenitors and 21 DIV neuronal cells was extracted using the reagent Trizol (RNAses inhibitor) according to the manufacturers' instructions (Invitrogen, Corp., California, USA). RNA samples' purity and concentration were determined in a spectrophotometer DeNovix. cDNA was obtained (500 ng RNA) by using Promega reverse transcriptase kit (Promega, Madison, USA). qRT-PCR reaction mixes (10 µL) consisted of cDNA (1 µL, 1:10), 200 nM of primers listed in Table 2 (Exxtend, Campinas, Brazil), 5 µL of the master mix Evagreen (Cellco, São Carlos, Brazil), and nuclease-free water. Quantitative real-time PCR was performed on a CFX384 Touch Real-Time PCR Detection System (Bio-Rad Laboratories, Inc., Hercules, CA, USA). Reactions followed an initial temperature increase of 50°C for 2 minutes and 95°C for 10 minutes, then 40 cycles at 95ºC for 15 seconds and 60ºC for 1 minute. Primers' specificities were assessed by a final melting curve, where samples were heated from 65ºC to 99ºC (1ºC increase every 5 seconds). Primer efficiencies varied from 95-120%. Data were normalized by the expression of internal reference genes ACTB (β-actin; 100 nM) and 18S (50 nM), and the relative quantification value for each target gene was analyzed using a comparative CT method (Vandesompele et al. 2002 Genome Biology, doi: 10.1186/gb-2002-3-7-research0034).

Table S5: Sequence of primers used for qRT-PCR

Gene	Forward primer	Reverse primer
**18S**	CCCAACTTCTTAGAGGGACAAG	CATCTAAGGGCATCACAGACC
**ACTB**	AGAGCTACGAGCTGCCTGAC	AGCACTGTGTTGGCGTACAG
**GAD65**	TGTACGGAGCATTTGACCCC	CATCAGTAATCCCCCACCCC
**GLS**	GCTGTGCTCCATTGAAGTGACT	TTGGGCAGAAACCACCATTAG
**MAP2**	CTCTCCTGTGTTAAGCGGAAA	AATACACTGGGAGCCAGAGC
**NMDAR1**	CTACCGCATACCCGTGCTG	GCATCATCTCAAACCACACGC
**NURR1**	CCACATAAACAAAGGCACATTGGC	CTGGACAGGCAAAAGGGACC
**PSD95**	AATGCCTACCTGAGTGACAGC	CATGGCTGTGGGGTAGTCGG
**SYP**	TTGTGAAGGTGCTGCAATGG	TCACTCTCGGTCTTGTTGGC
**TH**	AGTTCTCGCAGGACATTGGC	GCTTACACAGCCCGAACTCC
**TUBB3**	CAACCAGATCGGGGCCAAGTT	GAGGCACGTACTTGTGAGAAGA
**VGLUT1**	GGCTGTGTCATCTTCGTGAG	GCCCATTTGCTCCAGATCC

Supplementary Results

Table S6: Differentially expression of selected proteins for targeted Multiple Reaction Monitoring (MRM).

Accession	Gene Name	LogFC	p-value
**Organoids**			
Q04917	YWHAH	-1.5272	0.0359
P27816	MAP4	-1.2494	0.0998
P04216	THY1	-1.6637	0.0907
P35222	CTNNB1	-0.4241	0.2247
**21DIV Neurons**			
Q04917	YWHAH	0.3371	0.3288
P27816	MAP4	0.8543	0.0914
**NPC**			
Q04917	YWHAH	-0.5101	0.9329
P27816	MAP4	0.4135	0.4024

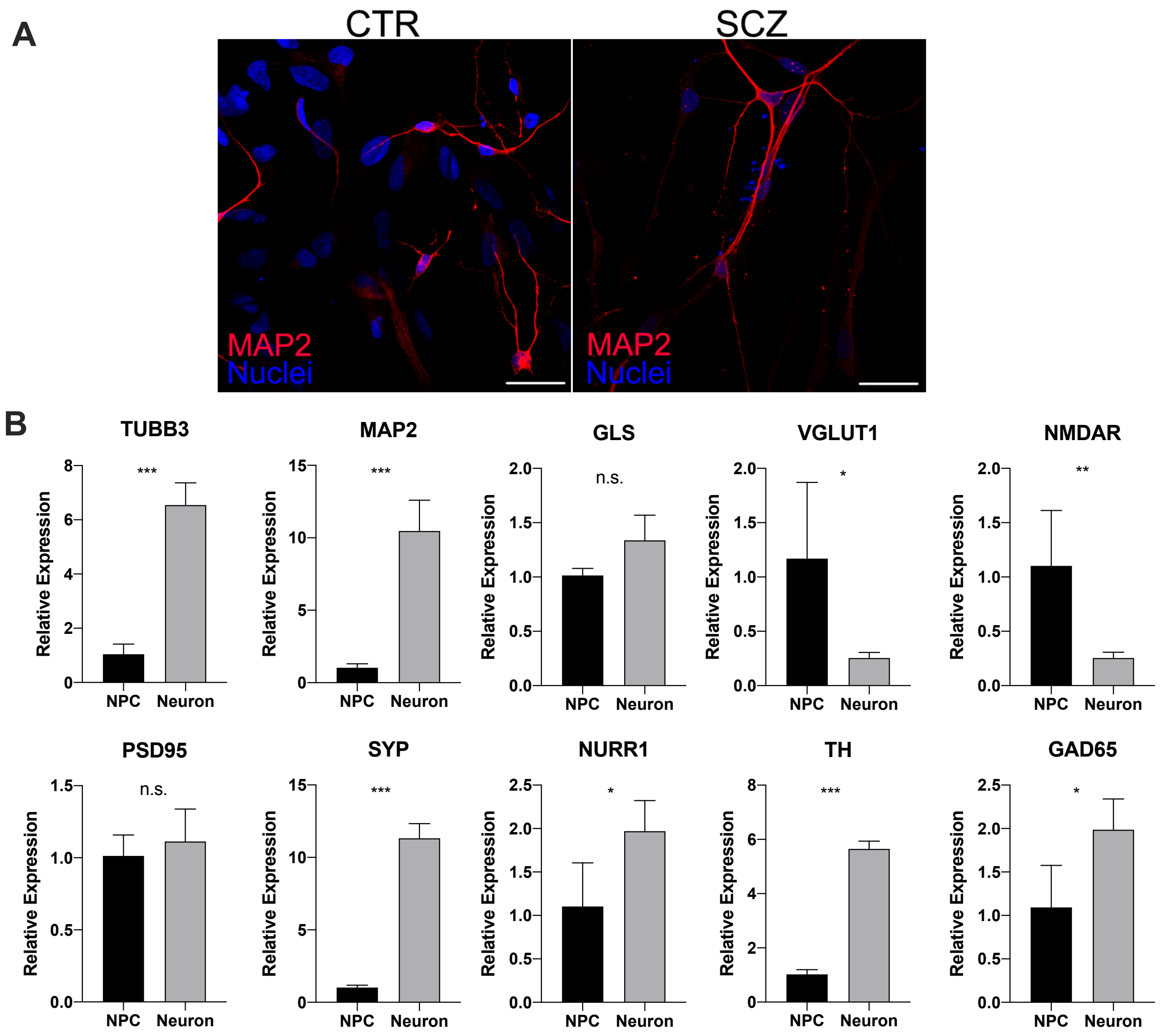

Figure S1: Characterization of neural differentiation. (A) Immunocytochemistry showing expression of MAP2 (red) in 21 DIV neurons. Representative micrographs of control (CTR) and schizophrenia (SCZ) cells. Scale bars = 30 µm. (B) Quantitative mRNA expression of progenitors and neurons (21 DIV). Tubulin b3 (TUBB3), microtubule-associated protein 2 (MAP2), glutaminase (GLS), glutamate transporter (VGLUT1, or SLC17A - solute carrier family 17 member 7), NMDA receptor (NMDAR1, or GRIN1 - glutamate ionotropic receptor NMDA type subunit 1), post-synaptic density protein 95 (PSD-95, or DLG4 - discs large MAGUK scaffold protein 4), synaptophysin (SYP), nuclear receptor related protein 1 (NURR1, or NR4A2 - nuclear receptor subfamily 4 group A member 2), tyrosine hydroxylase (TH), and glutamic acid decarboxylase (GAD65, or GAD2 - glutamate decarboxylase 2). Relative mRNA expression was calculated using ribosomal 18S and actin (ACTB) as reference genes, and average expression of progenitors were set as calibrators. Data is represented as mean ± SD (n=4), unpaired t-test *$p<0.05$, **$p<0.01$, ***$p<0.001$ and non-significant (n.s.).

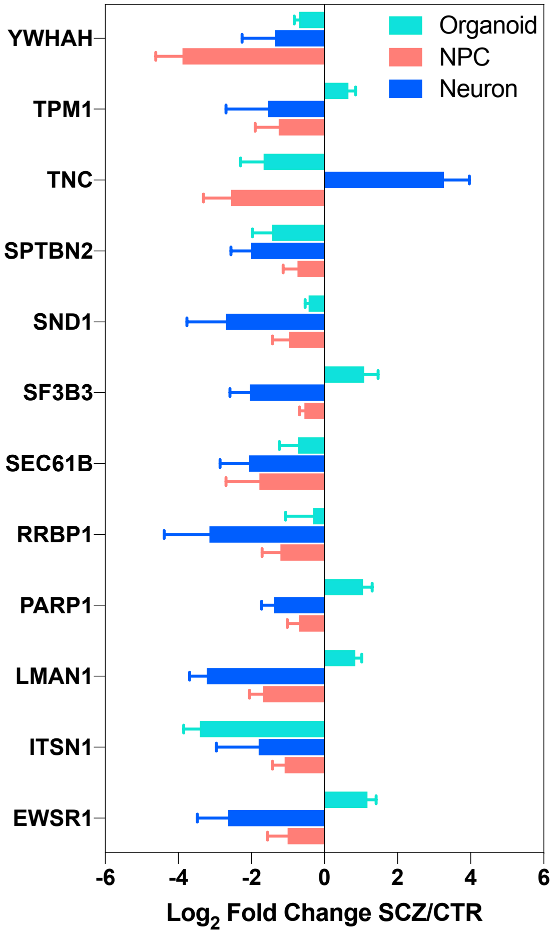

Figure S2: Differential regulation of proteins in schizophrenia patients versus control cells from human PSC-derived organoids, neurons, and NPCs. Proteins shown were found differentially regulated ($p<0.05$, ANOVA) in the three differentiation conditions. Data is represented as Log_2 Fold Change of schizophrenia versus control with SEM of replicates (n=9 for NPC and n=8 for Neurons and Organoids).

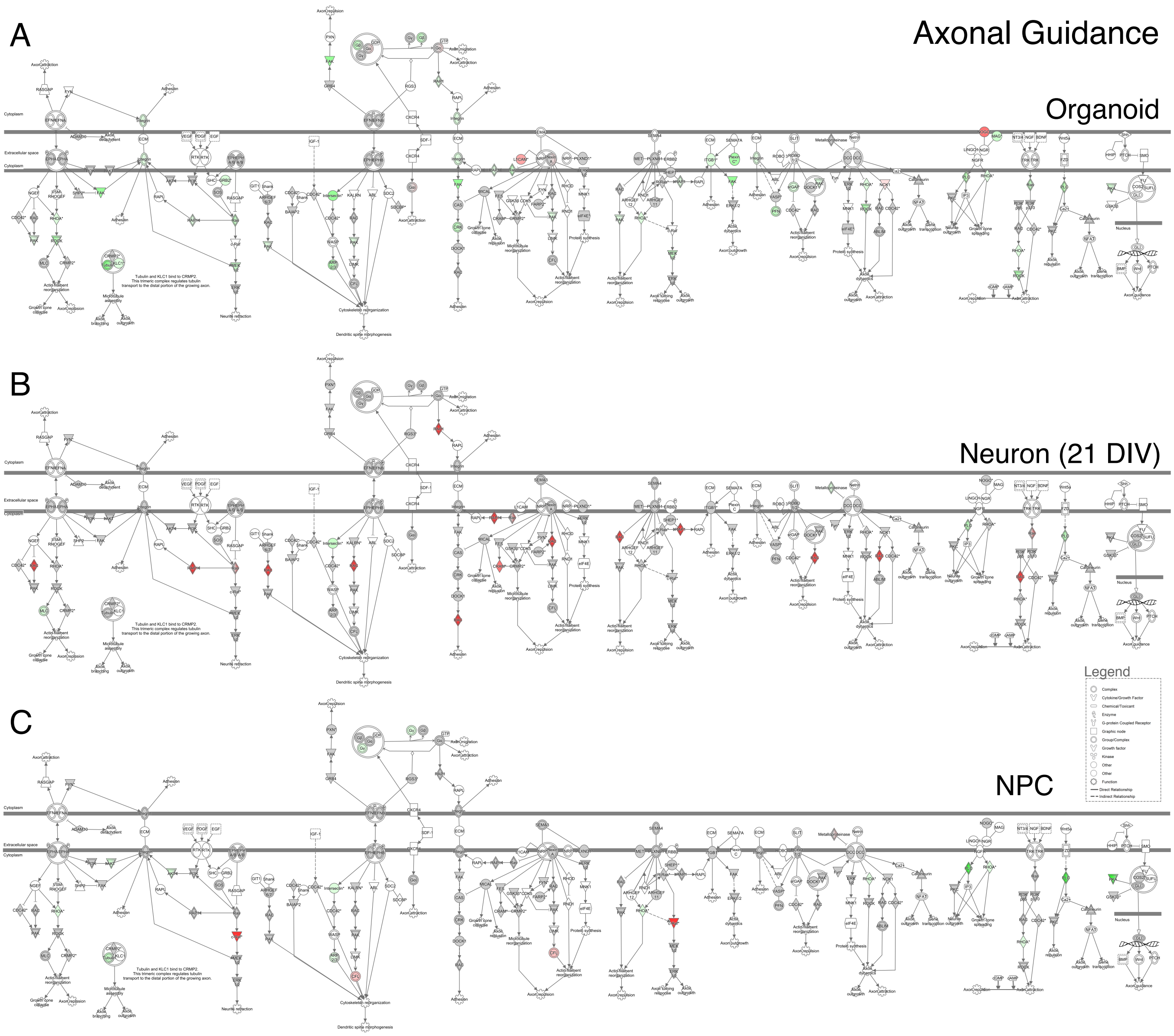

Figure S3: Canonical pathway Axon Guidance found in (A) organoids, (B) Neurons (21 DIV), and (C) NPCs, showing protein regulation according to IPA. Shades of red indicate protein upregulation and shades of green indicate protein downregulation. Uncolored symbols were not found in the proteome.

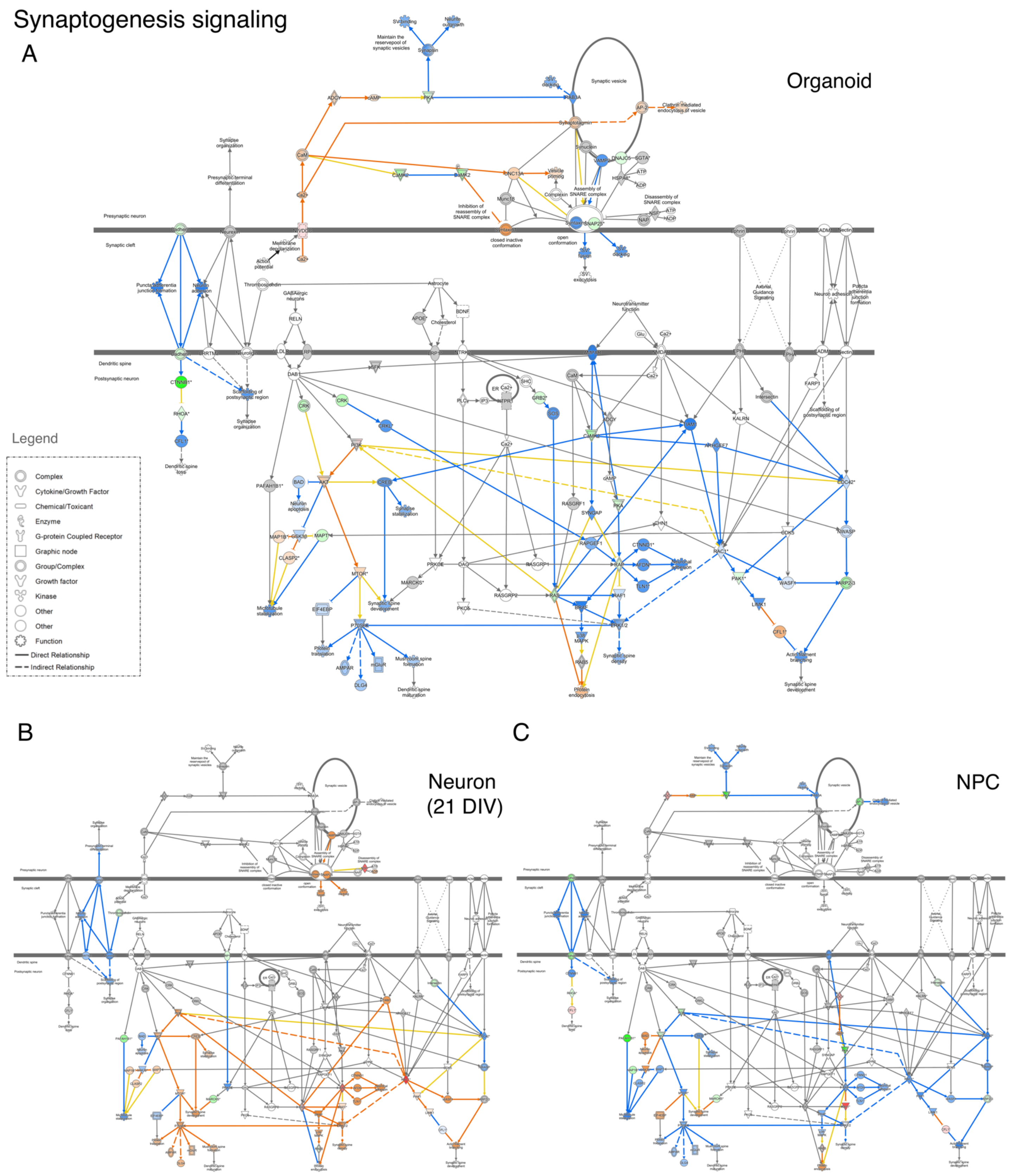

Figure S4: Synaptogenesis canonical pathway found in (A) organoids, (B) Neurons (21 DIV), and (C) NPCs, showing protein regulation according to IPA. Shades of red indicate protein upregulation and shades of green indicate protein downregulation. Pathway prediction state is represented by the z-score, with shades of orange indicating activation and blue inhibition of a protein or pathway (lines). Yellow lines indicate a divergent prediction to a protein regulation. Uncolored symbols were not found in the respective proteome.
